# Resolution of Inflammation: What Controls Its Onset?

**DOI:** 10.3389/fimmu.2016.00160

**Published:** 2016-04-26

**Authors:** Michelle A. Sugimoto, Lirlândia P. Sousa, Vanessa Pinho, Mauro Perretti, Mauro M. Teixeira

**Affiliations:** ^1^Laboratório de Sinalização Inflamação, Departamento de Análises Clínicas e Toxicológicas, Faculdade de Farmácia, Universidade Federal de Minas Gerais, Belo Horizonte, Brazil; ^2^Laboratório de Imunofarmacologia, Departamento de Bioquímica e Imunologia, Instituto de Ciências Biológicas, Universidade Federal de Minas Gerais, Belo Horizonte, Brazil; ^3^Laboratório de Resolução da Resposta Inflamatória, Departamento de Morfologia, Instituto de Ciências Biológicas, Universidade Federal de Minas Gerais, Belo Horizonte, Brazil; ^4^William Harvey Research Institute, Barts and The London School of Medicine, Queen Mary University of London, London, UK

**Keywords:** resolution, chemokine depletion, eicosanoids, pro-resolving mediators, tissue homeostasis

## Abstract

An effective resolution program may be able to prevent the progression from non-resolving acute inflammation to persistent chronic inflammation. It has now become evident that coordinated resolution programs initiate shortly after inflammatory responses begin. In this context, several mechanisms provide the fine-tuning of inflammation and create a favorable environment for the resolution phase to take place and for homeostasis to return. In this review, we focus on the events required for an effective transition from the proinflammatory phase to the onset and establishment of resolution. We suggest that several mediators that promote the inflammatory phase of inflammation can simultaneously initiate a program for active resolution. Indeed, several events enact a decrease in the local chemokine concentration, a reduction which is essential to inhibit further infiltration of neutrophils into the tissue. Interestingly, although neutrophils are cells that characteristically participate in the active phase of inflammation, they also contribute to the onset of resolution. Further understanding of the molecular mechanisms that initiate resolution may be instrumental to develop pro-resolution strategies to treat complex chronic inflammatory diseases, in humans. The efforts to develop strategies based on resolution of inflammation have shaped a new area of pharmacology referred to as “resolution pharmacology.”

## Introduction

Inflammation is a reaction of the host to infectious or sterile tissue damage and has the physiological purpose of restoring tissue homeostasis (1). However, uncontrolled or unresolved inflammation can lead to tissue damage, giving rise to a plethora of chronic inflammatory diseases, including metabolic syndromes and autoimmunity pathologies with eventual loss of organ function (2). In fact, signs of persistent unresolved inflammation are not only typical of classical inflammatory diseases but also an underlying feature of a variety of human conditions not previously thought to have an inflammatory component (3), including Alzheimer’s disease (4), atherosclerosis (5), cardiovascular disease (6), and cancer (7). This justifies the increasing interest in studying inflammatory processes. In this context, an important milestone has been reached with the awareness that engagement of resolution of acute inflammation is crucial to avoid persistent chronic inflammation and ensure proper return to homeostasis (8).

Historically, the first acknowledged report on resolution of inflammation was published in 1907 (9). This report shows that, in experimental irritant-induced pleurisy, a fluid containing fibrin and leukocytes was formed, disappearing after 5 days, with the clearance of “polynuclear leukocytes” and the persistence of mononuclear cells in the pleural cavity (9). For many years, resolution of inflammation was considered a passive phenomenon, merely associated with the removal of inflammatory stimuli, end of chemoattractant production, dilution of chemokine gradients over time, and prevention of further leukocyte recruitment. Some years later, the existence of endogenous inhibitors of leukocyte trafficking was reported, acting as a counteractive mechanism against promoters of cell recruitment, such as chemoattractants and adhesion molecules [reviewed in Ref. (10)]. Since then, several studies, especially those from Serhan’s lab at Harvard, showed that the resolution of inflammation is an active process brought about by the biosynthesis of active mediators, which act on key events of inflammation to promote the return to homeostasis (11–14). In this context, homeostasis is recovered after the production of pro-resolving mediators that act on specific receptor targets to (i) shutdown polymorphonuclear leukocyte recruitment, (ii) counteract signaling pathways associated with leukocyte survival to promote apoptosis (or programmed cell death), and (iii) activate the clearance of apoptotic cells (especially by macrophages through a non-phlogistic process), yielding (iv) macrophage reprogramming from a proinflammatory to a pro-resolving phenotype (15, 16).

**Figure 1:**
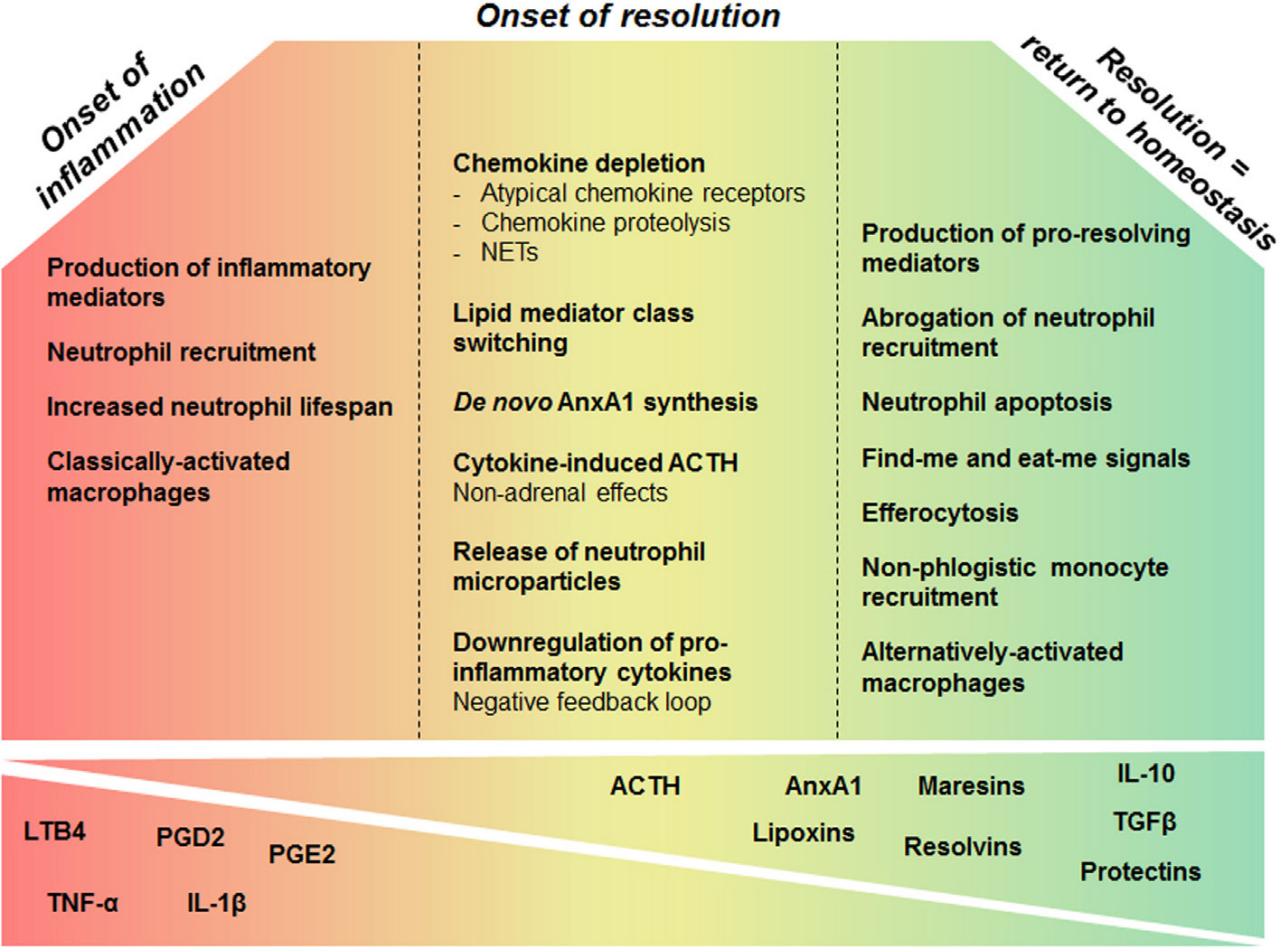
**Overview of cellular and molecular processes that govern inflammation and its resolution**. During early phase of inflammation, production of inflammatory mediators promotes leukocyte accumulation and survival in the inflammatory site. While the inflammatory response evolves, several mechanisms enable the fine-tuning of these phenomena creating a favorable environment for the resolution phase leading to return to tissue homeostasis. Chemokine proteolysis, sequestration by atypical receptors, and degradation by neutrophil extracellular traps (NETs) are important mechanisms to shape chemokine gradients restricting the influx of neutrophils, once sufficient numbers of cells have been recruited. In addition, inflammatory mediators may induce a negative-feedback loop downregulating the production of inflammatory cytokines. Prostaglandins generated in the active phase of inflammation are involved in the switch from proinflammatory lipid production to the synthesis of lipoxins and other pro-resolving lipids, within inflammatory exudates. Mediators released early in inflammation, like ACTH, can also enable the induction of the pro-resolving phase. Upon activation, neutrophils release microparticles containing pro-resolution mediators that control further granulocyte ingress and turn on a resolution and tissue reparative programs. AnxA1 is a major component of the pro-resolving properties of neutrophil-derived microvesicles. Many resolution mediators downregulate survival pathways and activate apoptosis of granulocytes. Apoptotic neutrophils release pro-resolving mediators that contribute to inhibition of continued neutrophil infiltration and to recruitment of monocytes in a non-phlogistic manner. Upon apoptosis, neutrophils also promote their own clearance by expressing find me and eat me signals that attract scavengers and allow the identification of the dying cell, respectively. In response to local mediators and upon efferocytosis, proinflammatory macrophages switch to resolution-phase macrophages. These events will reestablish tissue homeostasis.

Inadequate or insufficient resolution can lead to chronic inflammation, excessive tissue damage, and dysregulation of tissue healing, leading to fibrosis. Additionally, it has been implicated in multiple disease states, including the development of autoimmunity (2, 8, 17). Thus, understanding the mechanisms required for the resolution of inflammation may not only unveil new mechanisms of pathogenesis but also support the development of drugs that are able to manage inflammatory processes in directed and controlled ways. Resolution of inflammation requires pro-resolving molecular pathways that are triggered as part of the host response, during the inflammatory phase. This concept challenges a linear model of induction and resolution of inflammation, suggesting a more complex balance between proinflammatory and anti-inflammatory events that are initiated, at least partly, in parallel (18). The inflammatory cells involved in the active phase of inflammation undergo a functional repolarization and contribute to the onset of resolution. Additionally, an accumulating body of evidence suggests that many proinflammatory mediators that promote the inflammatory phase can simultaneously initiate a program for active resolution. For this reason, it is important to understand that adequate resolution of inflammation follows on a coordinated and florid proinflammatory phase with marked leukocyte accumulation. In this context, Sehran, who uncovered the most important pro-resolving lipid mediators, and Savill elegantly stated that “the beginning programs the end” meaning that the events occurring early in acute inflammation engage an active and coordinated “resolution program” (18). In this review, we reason on the events required for an effective transition from the proinflammatory phase to the onset and establishment of resolution (Figure 1).

## Cellular Events in the Resolution of Acute Inflammation

The molecular and cellular events of the inflammatory response are well known and typically characterized by increased blood flow, capillary dilatation, leukocyte infiltration, and production of chemical mediators. Acute inflammation is mainly characterized by the presence of neutrophils, which are highly motile leukocytes, able to rapidly migrate to the site of injury or infection. Although neutrophils are essential for proper elimination of the inflammogen, exaggerated influx of leukocytes can be more deleterious than the infection or injury itself and has been considered a bad marker of tissue homeostasis (19). Therefore, the key histological feature in the resolution of acute inflammation is the depletion of neutrophils from the local inflamed sites. This is achieved through programmed processes that occur in an overlapping fashion and are actively regulated at multiple levels (20, 21). The *cardinal signs of resolution* entail the limitation or cessation of blood-borne cell extravasation, the counter regulation of chemokines and cytokines, the switching off of signaling pathways associated with leukocyte survival, the induction of leukocyte apoptosis and their subsequent removal through efferocytosis by macrophages, the reprogramming of macrophages from classically activated to alternatively activated cells, the return of non-apoptotic cells to the vasculature or lymph, and finally the initiation of healing processes. Altogether, these events avoid excessive tissue damage and culminate in the return to tissue homeostasis, giving little opportunity for the development of chronic, non-resolving inflammation. On the other hand, failure of one or more steps in the resolution of inflammation may be involved in the pathogenesis of several human chronic inflammatory diseases (8).

## Pro-Resolving Mediators

Similar to the onset phase of inflammation, resolution of inflammation is coordinated and regulated by a large panel of mediators. The pioneer authors in the field of resolution and other investigators worldwide have focused on defining the endogenous mediators of resolution and the mechanisms through which the body regulates effector cells (PMNs, monocytes, and macrophages). It is worth noting that anti-inflammatory effects and pro-resolving effects are not totally overlapping: anti-inflammation mainly refers to an inhibitory/blocking action (e.g., stopping immune cell extravasation, which is a hallmark of acute inflammation), whereas pro-resolving actions indicate an inherent stimulation and activation of specific processes, such as apoptosis or efferocytosis. In both cases, the end point is the inhibition of inflammation, but pro-resolving mediators are those that genuinely enable resolution to take place (12, 22, 23). In the same vein, there is a mechanistic difference between an anti-inflammatory drug that blocks some specific pathways and a pro-resolving drug that is expected to activate a plethora of actions. Hence, the distinction is between blocking/inhibiting particular mediators, which can cause tissue damage, and agonism/activating cellular processes that participate in limiting or preventing damage, the latter enabling an amplifying effect. It is reasoned that pro-resolving-based therapies will promote both anti-inflammatory and pro-resolution actions, differing from traditional anti-inflammatory agents that solely inhibit key proinflammatory mediators (20). In addition, we have recently pointed out that pro-resolving molecules are characterized by “mild-to-moderate actions,” since they balance pro- and anti-inflammatory responses to reach an equilibrium (22).

According to the first consensus report from leading authorities on definitions and mechanisms in resolution (3) and subsequent reviews (16, 21), pro-resolving mediators should ideally fulfill some fundamental criteria that include:

- Stop: the limitation or cessation of neutrophil tissue infiltration;
- Sink: the counter regulation of chemokines and cytokines;
- Kill: the induction of apoptosis in spent neutrophils and their subsequent efferocytosis by macrophages;
- Skew: the reprogramming of macrophages from classically activated to alternatively activated cells;
- Leave: the return of non-apoptotic cells to the blood or lymphatic vasculature and egress of immune cells – following efferocytosis, the macrophages and dendritic cells leave the site of inflammation;
- Inform: the instruction of suppressive immune cells and adaptive immune response to help dealing with subsequent encounters;
- Heal: the induction of tissue repair – return to homeostasis without fibrosis or scar formation marks the final step of resolution.

Molecules that fulfill the criteria above, which qualify a pro-resolving mediator, are very diverse in nature (21) and include specialized lipid mediators [lipoxins (e.g., LXA_4_), resolvins (e.g., RvD1), protectins, and maresins] (14), proteins and peptides [e.g., annexin A1 (AnxA1), adrenocorticotropic hormone, chemerin peptides, and galectin-1] (24), gaseous mediators (e.g., H_2_S and CO) (25), a purine (adenosine) (26–28), as well as neuromodulators (acetylcholine and other neuropeptides) released under the control of the vagus nerve (29, 30).

Failure to produce adequate amounts of these anti-inflammatory and pro-resolving mediators or yet a failure to bind to their receptor could lead to the persistence of inflammation, playing a significant etiopathogenic role in chronic inflammatory and autoimmune diseases. This is highly plausible for inflammatory bowel diseases (IBDs), such as Crohn’s disease (CD) and ulcerative colitis (UC), chronic relapsing inflammatory conditions of the gastrointestinal tract that are characterized by intestinal inflammation and epithelial injury (31, 32). Resolution mediators (e.g., AnxA1, lipoxins, and resolvins) regulate intestinal mucosal injury, inflammation, and repair, supporting the resolution of inflammation in the gut. Therefore, defective expression of pro-resolution mediators may contribute to the chronic inflammatory response associated with IBD. Notably, colonic mucosa from UC patients demonstrates defective LXA_4_ biosynthesis, which may contribute to the inability of these patients to resolve persistent colonic inflammation (33). Complete loss of AnxA1 protein was detected in colonic tissues from chronic CD patients, which correlated with the clinical status, response to therapy, TNF-α expression, and lymphocyte activation (34). Vong and coworkers (35) documented an increase in mucosal synthesis of AnxA1 and LXA_4_, in individuals in medically induced remission from UC. Besides, during anti-TNF-α therapy, AnxA1 expression was upregulated in patients with a successful intervention, whereas non-responsive patients did not show the same expression profile (34). The contribution of AnxA1 to the remission of IBD was validated with a model of dextran sulfate sodium (DSS)-induced colitis in TNFR knockout (KO) mice, mimicking the anti-TNF-α therapy. Mucosal levels of AnxA1 increased in the absence of TNF-α signaling, allowing early recovery of colitis as compared to wild-type (WT) mice (36). According to these findings, changes in pro-resolving mediator levels may predict therapeutic efficacy. Moreover, inflammation-resolution agonists prevent immune-mediated tissue damage and restore tissue homeostasis. Interestingly, pharmacological treatment with LXA_4_ or Resolvin E1 (RvE1) effectively promoted the resolution of trinitrobenzenesulphonate (TNBS)-induced colitis (37, 38). The beneficial effect of lipid mediators in colitis was accompanied by decreased leukocyte infiltration and proinflammatory cytokines. In addition, TNBS-specific IgG serum levels decreased after treatment with RvE1, suggesting diminished antigen presentation and antibody production (38). Moreover, AnxA1 peptides encapsulated in nanoparticles accelerated the recovery of experimentally induced colitis and the healing of colonic biopsy-induced wounds (39).

Persistent airway inflammation in lung diseases, including asthma, may also be due to a defect in counter regulatory signaling (40, 41). Clinical findings suggest that severe asthma is associated with diminished expression of LXA_4_, its receptor FPR2, and 15-lipoxygenase, the major enzyme involved in LXs generation (42–46). Thus, LXA_4_-deficient production and/or signaling might have a role in the progression of the disease. In a recent study, AnxA1 and LXA_4_ plasma levels were lower in wheezy infants than in control group (47). Once persistent wheezing in children may progress to asthma, this reduced level of pro-resolving molecules could be an early event in asthma progression (48).

In some cases, failure in the activity of specific mediators may contribute to the inflammatory process even when the expression is normal or higher, when compared to healthy controls. For example, CD-related inflammation is characterized by reduced activity of the immunosuppressive cytokine transforming growth factor (TGF)-β1. TGF-β is a crucial cytokine in inflammation resolution due to its immunoregulatory activities, essential to tolerance and homeostasis, and its role in epithelial restitution and fibrosis (49). Indeed, *in vitro* and *in vivo* studies have demonstrated that TGF-β1 acts as a potent negative regulator of mucosal inflammation (50). Although TGF-β is found in high levels in human IBD tissue, it has reduced activity due to the overexpression of an inhibitor of TGF-β1 signaling, SMAD7 (51). As a result, TGF-β is unable to reduce the chronic production of proinflammatory cytokines that drives the inflammatory process in IBD and, consequently, inflammation is maintained (51). Notably, therapeutic strategies that restore TGF-β signaling pathway may downregulate the inflammatory response and induce remission in patients with CD (51, 52).

## Positive Networks in Resolution

Evidence is accumulating that a *pro-resolving cascade* becomes operative during resolution, whereby one pro-resolving mediator would induce another one. We reported one of the first evidence that fundamental pro-resolving mediators, such as AnxA1 and LXA_4_, induce the production of further anti-inflammatory molecules *in vivo*, such as IL-10 (53). Later, Brancaleone and colleagues (54) provided strong evidence that the engagement of FPR2/ALX by LXA_4_ induces AnxA1 phosphorylation and mobilization in human PMN. Similarly, the pro-resolving mediator RvE1 stimulates endogenous LXA_4_ production (55).

Other examples and *modus operandi* of this cross talk in resolution are emerging, as the cross talk between AnxA1 and glucocorticoid (GC)-induced leucine zipper (GILZ) during certain inflammatory events (56). GILZ mediate and mimic several anti-inflammatory actions of GCs (57). Besides demonstrating that GILZ expression depends on AnxA1, we identified that the lack of endogenous GILZ during the resolution of inflammation is compensated by AnxA1 overexpression. In the model of lipopolysaccharide (LPS)-induced pleurisy, GILZ deficiency was associated with an early increase of AnxA1 and equal neutrophil influx and resolution as compared to WT mice. Likewise, we demonstrated that dexamethasone-induced resolution was not altered in GILZ KO mice due to compensatory expression and action of AnxA1 (56). These studies indicate that pro-resolution mediators not only communicate in positive loops but also enact compensatory actions to guarantee the effective engagement of resolution pathways.

We predict that a further definition of the positive loops of resolution is crucial for the discovery of new pharmacological targets that could resolve inflammation, especially in the context of chronic inflammatory diseases. A better understanding of the key controlling points of resolution networks may allow us to design specific strategies to promote resolution.

## How Does Resolution Start?

Briefly, the acute inflammatory response can be divided in two stages: initiation (productive and transition phases) and resolution (Figure 1) (58). Interestingly, molecular and cellular mechanisms involved in the first phase of inflammation contribute to the initiation of the pro-resolving response. It has now become evident that coordinated programs of resolution initiate shortly after the beginning of the inflammatory response (18). In this context, several anti-inflammatory and pro-resolving mediators are endogenously produced to temper the inflammatory events. However, here we intend to highlight the existence of events and pathways that do not fulfill all criteria to be classified as pro-resolving, but do contribute to the initiation of resolution. These mechanisms provide the fine-tuning of inflammation, creating a favorable environment for the resolution phase to take place, and for homeostasis to return. As “contributors of resolution” these events, pathways, and mediators deserve special attention since they may be key targets for the pharmacological input or enacting of resolution, especially when it has not turned on, such as in chronic inflammatory settings.

Aside its well-known proinflammatory functions, nuclear factor kappa B (NF-κB) also has a crucial role in the initiation of resolution of inflammation. NF-κB proteins are a family of transcription factors of central importance in inflammation and immunity (59, 60). NF-κB and its activating IκB kinase (IKK)β play important roles in driving the inflammatory response by activating the expression of proinflammatory and anti-apoptotic genes (61). However, several reports have shown that NF-κB and IKKβ also influence anti-inflammatory response, pointing to their involvement in both onset and resolution of acute inflammation (62–64). The functional transcription factors consist in homo- or hetero-dimers comprising five subunits (p50, p52, p65, cRel, and RelB), which utilize Rel homology domain (RHD) for DNA binding and dimerization (65). Dimers containing at least one subunit with transactivating domains (TAD) in their C-terminus (p65, RelB, or cRel) are required to induce gene transcription. In contrast, dimers that contain only subunits without TAD (p50 and p52) are transcriptionally inactive and may prevent transcriptionally active NF-κB dimers from binding to κB sites (66). In resting cells, NF-κB dimers are sequestered to the cytoplasm and maintained inactivated by reversible association with its inhibitor IκB or unprocessed forms of cytoplasmic p50/p105 (NF-κB1) and p52/p100 (NF-κB2) (60, 65, 67). NF-κB activation in response to proinflammatory stimuli is regulated by IKK, which phosphorylates IκB and promotes its proteasome degradation and the release of NF-κB for nuclear translocation and gene transcription activation (61).

Nuclear factor kappa B activates many promoters containing highly divergent κB-site sequences. The fact that the regulation of gene expression is dimer-specific explains, in part, how NF-κB pathways can modulate both inflammation and resolution (65, 68). Differential expression of NF-κB subunits and the differential effects of NF-κB dimers may be intimately associated with the temporal regulation of inflammatory responses (69). p65/p50 heterodimer is the predominant form of functionally active NF-κB with proinflammatory activity, since this dimer enhances the transcription of genes related to the proinflammatory phase. On the other hand, p50/cRel, p65/cRel, or p50/p50 seems to be involved in the transcription of genes related to the recovery phase (70). Accordingly, the genes regulated by p50/cRel and p65/cRel are activated in later points after inflammatory stimulation, providing the necessary period between the burst of the proinflammatory response and the recovery phase (69, 71). p50/p50 homodimer exerts important anti-inflammatory and pro-resolving effects and competes with p65/50 heterodimer for DNA binding (72, 73). Unlike p65/p50, p50/p50 lacks the transactivation domain and may repress proinflammatory genes (74–76). Bohuslav and colleagues demonstrated that increased expression of p50 subunit of NF-κB directly results in the downregulation of LPS-induced TNF production (72). Recently, the enhancement of efferocytosis mediated by RvD1 was associated with p50/p50-mediated suppression of TNF-α expression (77). In this context, RvD1 modulates at least two different NF-κB pathways leading to enhanced localization of p50 in the nucleus, while it suppresses dissociation from IκBα and concurrent nuclear translocation of p65 (77). Moreover, upon LPS stimulation, macrophages express p65/p50 heterodimer in predominance over p50/50 homodimer, thereby provoking the proinflammatory state. However, in later time points, these macrophages show p105 degradation, nuclear translocation of p50, and formation of p50/p50 homodimer, presumably as an adaptive cellular response to proinflammatory insult.

During the proinflammatory phase, besides inducing proinflammatory genes, p65/p50 also induces the transcription of genes that will provide the control of the recovery phase, such as Rel, the gene that codifies cRel (71). For example, Muxel and colleagues showed that the expression of p65/cRel, crucial for inflammation resolution, is induced by p65/p50, which is earlier expressed in LPS-stimulated macrophages (69). The authors identified that temporal regulation of cRel promoted the synthesis of melatonin (*via* p65/cRel) by macrophages, a modulator of phagocyte function preventing over-activation of this cell type (78, 79). In addition, NF-κB negatively regulates NLRP3-inflammasome activation and IL-1β production (63). In macrophages, NF-κB prevents premature and excessive NLRP3-inflammasome activation, acting as a negative regulator of IL-1β secretion (63). Although the precise molecular mechanism underlying NF-κB-mediated inhibition of NLRP3-inflammasome activation remains unclear, NF-κB has been suggested to promote autophagy (80), a cellular process that negatively regulates NLRP3 inflammasome activity (81–83). Reinforcing this observation, a recent study revealed that NF-κB restricts inflammasome activation in macrophages *via* elimination of damaged mitochondria (84). This allows NF-κB to restrain its own inflammation-promoting activity in macrophages (84).

Clearly, NF-κB may have dual function in inflammation, which is likely the result of the central role of this molecule in the convergence of several inflammatory signals (62). This results in divergent effects of NF-κB pharmacological inhibition in inflammatory models. On the one hand, NF-κB inhibitors may attenuate inflammation and promote resolution in different experimental models of inflammation (62). For example, NF-κB inhibitors possess anti-inflammatory effects in models of LPS-induced lung injury (85), traumatic brain injury (86), colitis (87), and pulmonary arterial hypertension (88). Our research group showed that inhibition of NF-κB promotes resolution in established murine models of neutrophilic and eosinophilic inflammation associated with enhanced apoptosis of inflammatory cells (89, 90). On the other hand, inhibition of NF-κB during the resolution of inflammation prolonged the inflammatory response and prevented apoptosis (62). In addition, IKKβ has also been shown to have an anti-inflammatory role, such as the suppression of M1 macrophage activation during infection through the inhibition of signal transducer and activator of transcription (STAT)1 pathway (91). In accordance with this observation, IKKβ ablation results in severe neutrophilia and inflammation mediated by IL-1β (92). Notably, mice lacking IKKβ had hyperproliferative granulocyte–macrophage progenitors and pregranulocytes and a prolonged lifespan of mature neutrophils that correlated with the induction of genes encoding pro-survival molecules (92). Of clinical relevance, enhanced inflammation and neutrophilia were observed in human subjects that were treated with IKKβ inhibitors.

Notably, proinflammatory and resolution phases of inflammation are under the control of both transcriptional and post-transcriptional mechanisms, which regulate the expression of proteins that initiate and resolve inflammation. Reviewing this topic in 2010, Anderson (93) pointed out that post-transcriptional controlling mechanisms link the initiation/productive phase to the resolution phase of inflammation. mRNA translation is a highly regulated process governed by post-transcriptional mechanisms. Transcription is the first step in the regulation of gene expression, but since mRNA can be long-lived, turning off its synthesis does not rapidly redirect or stop the progress of inflammation. On the other hand, the second step, i.e., post-transcriptional regulation, can rapidly suppress protein expression by promoting mRNA degradation or by inhibiting its translation (93). Post-transcriptional control mechanisms may rapidly limit the expression of potentially toxic inflammatory mediators and help protecting the host against the pathological overexpression of potentially injurious proteins. For instance, a number of cytokine mRNAs can be regulated at the level of mRNA stability (94). mRNA decay and translational repression of target transcripts are promoted by RNA-induced silencing complex (RISC) that is composed by argonaute proteins bound to small non-coding RNAs, microRNAs (miRNAs). Importantly, the mechanisms used to ensure limited production of the proteins involved in the inflammatory response are highly variable, and in some cases, interact with each other to define protein expression levels. It remains not fully understood whether post-transcriptional controlling mechanisms play a role in the resolution of inflammation, but exciting possibilities for pharmacological intervention against the overproduction of many inflammatory proteins are likely to emerge from this elucidation (95).

Importantly, miRNAs triggered by immune mediators have a central role in modulating NF-κB signaling pathways and might be involved in controlling the switch from a strong early-inflammatory response to the resolution phase of the inflammatory process, in a timely and orchestrated manner (96, 97). The endotoxin-responsive gene miR-146a was the first one to be discovered to suppress the activation of the NF-κB pathway (98). miR-146a has been described as a negative regulator of the canonical NF-κB inflammatory cascade by targeting IL-1 receptor-associated kinase (IRAK) 1 and TNF receptor-associated factor (TRAF) 6 (98, 99). Moreover, miR-146a targets RelB, which is mostly implicated in the non-canonical NF-κB pathway, and controls monocyte responses during inflammatory challenge (100, 101). Some studies indicate that miR-146a can regulate proinflammatory gene expression by controlling RelB-dependent reversible chromatin remodeling (102, 103). Notably, deletion of miR-146a gene results in the production of higher levels of inflammatory cytokines by macrophages (104). Remarkably, the expression of many miRNAs is induced in an NF-κB-dependent manner after inflammatory stimulus or pathogen infection, promoting the control of the strength and longevity of an inflammatory response (97, 98, 104–109). miR-146a was the first reported miRNA whose expression can be induced through the NF-κB-dependent pathway in response to various immune mediators, such as LPS, IL-1β, and TNF-α (98, 105, 110–113). Since then, many studies have further identified subsets of miRNAs related to the TLR-induced NF-κB-dependent pathway. Another example, miR-9 expression is directly induced by LPS *via* the TLR4-MyD88-NF-κB-dependent pathway in human monocytes and neutrophils. In turn, miR-9 operates a feedback control of the NF-κB-dependent responses by fine-tuning NF-κB1 expression. Bazzoni and colleagues suggest that miR-9 induction probably acts as a tuning mechanism to prevent negative regulation by p50 homodimers, as occurs in monocytes in systemic anti-inflammatory response syndrome (SIRS) (109).

Because miRNA-mediated post-transcriptional control is important to fine-tune the expression of genes involved in inflammation, dysregulation of expression levels of miRNAs can lead to chronic infections, autoimmunity, allergic inflammation, or immune deficiency. Recent studies have identified dysregulated miRNAs in tissue samples of IBD patients and have demonstrated similar differences in circulating miRNAs in the serum of these patients [reviewed in Ref. (114)]. In fact, dysregulated expression of tissue and blood miRNAs in IBD already numbers >100 (114) and may be involved in the reduced apoptosis of T-cells, which is an important mechanism in T-cell homeostasis, and cell activation (115).

Also important for resolution initiation, the pituitary hormone adrenocorticotrophin (ACTH) is released quite early during inflammation, in response to proinflammatory cytokines, including IL-1β (116). For a long time, ACTH has only been thought to modulate host response through the rapid generation of adrenal-derived GCs, which are *de novo* synthesized from cholesterol. However, recent works have revealed important immune-modulatory properties of ACTH, through the activation of specific receptors in the periphery (117), expressed on macrophages and other stromal cells such as chondrocytes [reviewed by Montero-Melendez (118)]. Molecules that activate these receptors on macrophages are able to promote resolution of inflammation with a downstream impact on experimental arthritis (119, 120).

### Chemokine Depletion Decreases Infiltration of Neutrophils into Tissue

As discussed above, successful inflammation depends on the regulation of neutrophil recruitment, allowing the proper elimination of the inflammogen but avoiding the tissue damage induced by excessive neutrophil influx and toxic content release. According to Headland and Norling – who recently reviewed this subject (21) – restricting the influx of neutrophils, once sufficient number of cells has been recruited, is a process through which chemokine and cytokine gradients are reduced, proinflammatory lipid mediators are switched to pro-resolving mediators, and circulating neutrophils are no longer activated and recruited to the inflammatory site. Chemokines are low molecular weight cytokines that orchestrate the migration of target cells to the site of inflammation. Chemokine depletion through mechanisms, such as chemokine cleavage by proteolysis and chemokine sequestration, is necessary to achieve a resolving environment and to abrogate neutrophil influx (16). Chemokines directly induce cell migration through a set of conventional chemokine G protein-coupled receptors. However, chemokines are also recognized by a small subfamily of atypical chemokine receptors (ACKR), previously called decoys, interceptors, scavengers, or chemokine-binding proteins (121). The binding of chemokines to their respective atypical receptors does not promote leukocyte migration due to the inability of ACKR to initiate classic G protein-dependent signaling pathways. Instead, ACKR sequestrate chemokines from the environment, an important mechanism to shape chemokine gradients. Therefore, ACKR are now emerging as crucial regulatory components of chemokine networks in a wide range of physiologic and pathologic contexts (122).

Chemokine proteolysis is another important mechanism for chemokine depletion and consequently the decrease of neutrophil recruitment and activation. Matrix metalloproteinases (MMPs) are traditionally associated with extracellular matrix protein degradation in several physiological and pathological processes. However, it is now clear that MMPs mediate homeostasis of the extracellular environment by modulating the biological activity of many bioactive molecules involved in cell function (123, 124) and innate immunity (125), including chemokines (123, 126–130), TNF-α (124, 131), α-defensin (132), and mannose-binding lectin (133). In this context, Dean and colleagues (134) proposed that macrophages aid the regulation of acute inflammatory responses by precise proteolysis of chemokines through MMP-12. Macrophage-specific MMP-12 cleaves CXC chemokines in the ELR motif, which is fundamental for receptor binding, thus rendering the mediators unable to recruit neutrophils (134). In some cases, cleaved chemokines continue to bind to their corresponding receptors, but fail to induce downstream signaling and chemotaxis, thus acting as antagonists dampening inflammation (126, 127).

### Pro- and Anti-Inflammatory Networks Help to Turn on the Resolution Program

A great number of evidence indicates that proinflammatory molecules can be involved in the initiation of the resolution program. In order to limit the undesirable consequences of an excessive inflammatory process, many mediators involved in the onset of the inflammatory response simultaneously trigger a program that actively resolves inflammation. In this context, our group has observed, in two complementary studies, the intricate balance and cross talk between pro- and anti-inflammatory cytokines during a systemic inflammatory response. In 2003, we described a network of TNF-α, IL-1β, and IL-10 during severe intestinal ischemia and reperfusion injury (135). Both, IL-1β and TNF-α triggered an anti-inflammatory cascade resulting in the production of IL-10. We identified that IL-1β plays a major role in driving endogenous IL-10 production and protecting against TNF-α-dependent systemic and local acute inflammatory response. IL-1β has been implicated in inflammatory events, such as the expression of adhesion molecules and neutrophil influx following reperfusion of ischemic tissues. However, some studies have failed to show a protective effect of IL-1β inhibition during ischemia/reperfusion (I/R) injury (136–138). In our investigations, we associated neutralizing strategies or selective receptor antagonism to prevent the actions of IL-1β with an overall enhancement of tissue injury, proinflammatory cytokine expression (TNF-α), and lethality (135). Members of the IL-1 family of cytokines (e.g., IL-1β, IL-18, and IL-36γ) display a dual role in regulating IBD, reinforcing the concept that proinflammatory cytokines may contribute to both proinflammatory responses and resolution of inflammation. These cytokines are upregulated in the inflamed mucosa during experimental colitis as well as in human IBD. Remarkably, they not only contribute to intestinal inflammation (139) but also to resolution of inflammation, as demonstrated by the increased susceptibility to DSS-induced colitis by mice lacking IL-1β, IL-18, and IL-36 receptors or components of their processing (140–144). In humans, polymorphisms leading to decreased Nlrp3 expression, and consequent hypoproduction of IL-1β, are associated with increased risk of developing CD (145).

Moreover, we and others have observed that TNF-α is central to the pathogenesis of reperfusion-associated injury and lethality (135, 146, 147). However, this proinflammatory cytokine also contributes to the production of IL-10 during intestinal ischemia and reperfusion (147). Furthermore, we reported that TNF-α modulates IL-1β production: first, inhibition of TNF-α was accompanied by enhanced reperfusion-induced production of IL-1β (147); second, administration of exogenous IL-10 was linked to decreased TNF-α concentration and enhanced IL-1β. Based on these results, we hypothesized that TNF-α could be inducing an intermediate molecule that controls IL-1β production (147). It is interesting to note that recent investigations have identified a central role for TNF-α in upregulating a pro-resolving master receptor that transduces the actions of AnxA1, LXA_4_, and RvD1 (148).

Several studies have identified a mechanism feedback for IL-10 as a potent repressor of proinflammatory cytokine production by macrophages, acting therefore as a key anti-inflammatory mediator (149, 150). In murine bone marrow-derived macrophages (BMDM) activated by LPS, IL-10 attenuated proinflammatory cytokine production *via* reduction of mRNA stability. IL-10 initiates a STAT3-dependent increase of the expression of the RNA destabilizing factor tristetraprolin (TTP) accompanied by the release from p38 MAPK-mediated inhibition. As a result, IL-10 diminishes mRNA and protein levels of TNF-α and IL-1β (151).

### Resolution of Inflammation Is Accompanied by an Active Switch in the Mediators That Predominate in Exudates

In a classical acute inflammatory response, proinflammatory lipid mediators, such as the classical eicosanoids [prostaglandins (PGs) and leukotrienes (LTs)], are generated during the initial phase of the inflammatory response through enzymatic modification of arachidonic acid (AA) by cyclooxygenases (COX) and lipoxygenases (LO) (152). These proinflammatory molecules have important roles in initiating leukocyte trafficking and stimulating blood flow changes, increasing vasopermeability to yield edema formation, all leading to neutrophil influx to the site of inflammation (14). In addition, PGs and LTB_4_ are involved in the initiating steps that permit leukocytes to leave postcapillary venules *via* diapedesis (153). Thereby, a switch in lipid mediators from proinflammatory PGs to lipoxins, which are anti-inflammatory/pro-resolving mediators, is crucial for the transition from inflammation to resolution (154). As Serhan pointed out in a scholar review (20), during inflammation, neutrophils undergo a phenotype switch to produce different profiles of lipid mediators depending on the cells and substrates present in the local environment. Neutrophils in the peripheral blood generate and release LTB_4_ on activation, as one of their main bioactive products. During spontaneous resolution of acute inflammation, there is a switch in PMN-LO pathway products expression, from LTs to lipoxins and resolvins. Evidence indicates that first-phase proinflammatory eicosanoids “reprogram” the exudate PMN to produce pro-resolving lipid mediators and hence promote resolution. For instance, Levy and colleagues suggested that when circulating PMNs begin diapedesis, they are exposed to autacoid gradients (e.g., PGE_2_) that initiate phenotypic changes *via* gene expression regulation (12). In this context, local PGE_2_ and PGD_2_ stimulate the processing of 15-LO mRNA in leukocytes to produce functional enzymes for the synthesis of lipoxin. AA is then converted to anti-inflammatory lipid mediators, such as LXs (e.g., lipoxin A_4_ and lipoxin B_2_), which harness dual anti-inflammatory and pro-resolving actions, *in vitro* and *in vivo* (20). Lipoxins are generated by transcellular biosynthesis, involving two or more cell types, since the required enzymes are differentially expressed in the cells. Thus, at the sites of injury or inflammation, LXs are generated *via* biosynthetic routes engaged during cell–cell interactions. Mobilization of LX biosynthetic circuit occurs, for example, when infiltrating PMNs (which express 5-LO) interact with tissue resident cells (which express 15-LO) in inflamed target organs. In an autocrine, paracrine, or juxtacrine manner, newly formed LXs can interact with specific receptors on leukocytes to regulate their function (12).

Cyclooxygenase-2 apparently has a dual role in the inflammatory process, initially contributing to the onset of inflammation and later helping to resolve the process. Gilroy and colleagues reported that COX-2 expression and PGE_2_ levels transiently increased in the early stage of carrageenan-induced pleurisy in rats (155). Later in the response, COX-2 was induced again to even greater levels and generated anti-inflammatory PGs, such as PGD_2_ and 15-deoxy-Delta(12,14)-PGJ_2_ (15d-PGJ_2_), but only low levels of proinflammatory PGE_2_. Anti-inflammatory actions mediated by 15d-PGJ_2_, a terminal product of COX-2 pathway, represent another negative feedback that explains how once-initiated immunologic and inflammatory responses are switched off and terminated. 15d-PGJ_2_, a terminal product of COX-2 pathway, is abundantly produced in inflamed sites, suggesting its potential role in facilitating the resolution of inflammation (156). 15d-PGJ_2_ exerts potent anti-inflammatory actions, in part by antagonizing the activities of NF-κB, STAT3, and activator protein 1 (AP1), while stimulating the anti-inflammatory nuclear factor E2-related factor 2 (Nrf2). Besides targeting the transcriptional machinery, 15d-PGJ_2_ is a potent inhibitor of protein translation. Interestingly, 15d-PGJ_2_-mediated translational repression triggers a stress response program that results in the assembly of stress granules containing untranslated mRNAs. Stress granules have an important role in reprogramming gene expression to allow stressed cells to survive to noxious stimuli (157, 158). Altogether, these mechanisms might combine to effectively dampen inflammation (93). Thus, 15d-PGJ_2_, especially formed during the late phase of inflammation, might inhibit cytokine secretion and other events by antigen-presenting cells such as dendritic cells or macrophages. Production of the 15d-PGJ_2_ is a consequence of a series of dehydration (oxidation) of PGD_2_ (159). The latter is a major COX-2 product formed in various cells (e.g., mast cells) and tissues during inflammatory processes by the action of PGD_2_ synthase, which catalyzes the isomeric conversion of PGH_2_ to PGD_2_. The pathogenic relevance of PGJ_2_ is suggested by clinical findings of reduced levels of PGD_2_ in some human diseases, such as the cerebrospinal fluid of patients suffering from multiple sclerosis and schizophrenia (160). Other evidence of clinical relevance comes from atherosclerosis, where PGE_2_ is over-expressed in symptomatic plaques of patients who underwent carotid endoarterectomy, while in asymptomatic ones, the PGD_2_ pathway prevails, known to be associated with NF-κB inactivation and MMP-9 suppression. These clinical findings suggest that PGE_2_-dominated eicosanoid profile is associated with cerebral ischemic syndromes, possibly through MMP-induced plaque rupture (161).

Although therapeutic inhibition of COX-2 by non-steroidal anti-inflammatory drugs (NSAIDs) may have beneficial effects in the early phase of inflammation by preventing prostanoid production, it may also be “resolution-toxic,” by disrupting the production of anti-inflammatory PGs and LXs (3, 155, 162, 163). Disturbance of physiologic lipid mediator class switching by COX-2 inhibitors has deleterious consequences in humans (164) as well as in murine peritonitis (163), arthritis (165), and lung acute injury (ALI) models (166). In the study from Fukunaga and colleagues, COX-2 inhibition resulted in an exacerbation of ALI with longer recovery times. Reinforcing the dual role of COX-2 during inflammation, inhibition of COX-2 activity by pharmacologic treatment or gene targeting decreased early PMN trafficking to the lung but paradoxically led to dramatic increases in inflammation at later time points, mainly due to the disruption of LXA_4_ production (166). Furthermore, COX-2 inhibition decreased macrophage phagocytosis of apoptotic PMNs *in vitro* and reduced prostaglandin E2 and LXA_4_ expression (163). During peritonitis, treatment with specialized pro-resolving lipid mediators [aspirin (ASA)-triggered lipoxins, RvE1, and protectin D1] rescued the resolution deficit promoted by COX-2 inhibition (163).

Aspirin is unique among other NSAIDs because it irreversibly inhibits COX-2 by acetylation of an amino acid serine residue preventing prostanoid generation (167) yet enabling the biosynthesis of endogenous anti-inflammatory mediators. Therefore, the generation of ASA-triggered specialized lipid mediators (AT-SLM) (11, 168–170) may enhance resolution and counteract the loss in prostaglandin production by ASA (18). Low-dose ASA triggers the resolution phase by activating endogenous epimers of specialized pro-resolving lipid mediators in humans and several animal models (3). Low-dose ASA triggers 15-epi-LXA_4_ in skin blisters in humans to reduce PMN infiltration by inducing antiadhesive nitric oxide, thereby dampening leukocyte/endothelial cell interaction and subsequent extravascular leukocyte migration (171). In addition, low-dose ASA administration to mice triggered the formation of 15-epi-LXA_4_, which in turn attenuated I/R-mediated vascular inflammation (172). In a randomized controlled study, low-dose ASA administration to volunteers augmented plasma ATL levels while inhibiting thromboxane (173). These observations support the idea that low-dose ASA may be considered “resolution friendly” (18), since it mimics endogenous biosynthetic mechanisms to trigger new mediators, leading to a favorable net change (173) for pro-resolution (174, 175).

### Neutrophils: Important Cells to Turn on Resolution

Aborted neutrophil recruitment is one of the steps required to reconstitute tissue homeostasis, followed by apoptosis and clearance by macrophages. Interestingly, neutrophils have pivotal roles in attenuating inflammatory diseases and seem to orchestrate both elimination of microorganisms and resolution of inflammation (21). In view of that, wound healing is delayed in neutrophil depletion models, indicating a critical role of these cells in the resolution of inflammation (176). Moreover, depletion of neutrophils aggravates different types of experimental UC (177, 178) and extends joint inflammation in a murine model of gout (179). Among the anti-inflammatory functions of neutrophils, it is worth mentioning its capacity to disrupt chemokine gradients *via* several mechanisms. For instance, neutrophils release proteases that not only degrade extracellular matrices and cells surrounding the inflammatory milieu but also deactivate inflammatory cytokines (180). Additionally, neutrophils modulate the cytokine production stimulated by bacterial peptidoglycans and LPS (181). *In vitro* studies have shown that PMN lysates and neutrophil elastase can degrade recombinant human IL-1β and TNF-α but not IL-10, and alpha1-antitrypsin can inhibit this process (180). Neutrophil-derived proteases are also involved in the downregulation of IL-1β and TNF-α produced by mononuclear cells, an effect that is independent on ROS production or phagocytosis (180). Serine proteases released by activated neutrophils may also be associated with NETs, which are web-like structures composed of nuclear material in complex with neutrophil proteins that display exquisite antibacterial properties (182). A recent article by Schauer and colleagues revealed that at the very high neutrophil densities that occur at the site of inflammation, NETs build aggregates that trap and degrade proinflammatory mediators *via* the proteolytic action of inherent neutrophil serine proteases (179). However, it remains to be investigated if this anti-inflammatory effect can be reproduced in physiological conditions where concentrations of NET may be lower. Conversely, NETs are also related to proinflammatory effects that in part induce further neutrophil recruitment (183). Recent observations suggest that NETs are effective activators of the inflammasome machinery in both human and murine macrophages, resulting in the release of active IL-1β and IL-18 (184). Indeed, pharmacological and genetic strategies that prevent NETosis have been shown to be protective in murine models of lupus (185), cardiac infarction (186), deep vein thrombosis (187), atherosclerosis (183), and diabetes (188). In addition, a recent work suggests that damage-associated molecular patterns (DAMPs) released during liver I/R result in formation of NETs which subsequently exacerbate organ damage and initiate inflammatory responses (189). Moreover, the presence of DNAse-sensitive NETs in skin wounds impairs wound healing in diabetes (188). Timely degradation/removal of NETs is critical since its components may serve as autoantigen or DAMPs leading to inflammatory and chronic autoimmune diseases, including systemic lupus erythematosus (SLE) [reviewed in Ref. (189–194)]. Furthermore, mitochondrial ROS-dependent NETosis may promotes externalization of proinflammatory oxidized mtDNA and subsequent activation of type I interferon (IFN) synthesis, what may contribute to lupus-like disease (195). Finally, serum of SLE patients show an increase in various NET proteins [e.g., defensins, high-mobility group box protein 1 (HMGB1), and bactericidal proteins] compared to healthy-donor blood, indicating that NETosis may be implicated in the genesis and/or amplification of the disease (196, 197). Therefore, like uncleaned apoptotic and necrotic cell remnants, uncleaned NETs may contribute to inflammation and autoimmunity.

Another neutrophil-related mechanism that is worth mentioning here is the release of S100A8 and S100A9 proteins and their calprotectin heterocomplex, upon stimulation. These proteins have been shown to have dual biological functions on inflammation (198–201). Abundant in neutrophils, calprotectin is released at sites of infection where it exerts antimicrobial activity, which is attributed to its ability to chelate manganese and zinc (200–205). In addition, calprotectin activates the innate immune system through activation of the receptor of advanced glycation end products (RAGE) and TLR4, resulting in downstream NF-κB activation and secretion of proinflammatory cytokines, such as TNF-α and IL-17 (206–208). Diverging properties of calprotectin related to PMN recruitment and functions have been described. Calprotectin was shown to activate the recruitment of PMNs and stimulate their adhesion by activating MAC-1 β2 integrin (209). Moreover, the functional blockage of calprotectin reduced PMN recruitment stimulated by LPS *in vivo* (210). Conversely, studies have pointed to the ability of S100A8 and S100A9 to repel PMNs (fugetaxis) and inhibit their chemotaxis toward chemokines *in vitro*. Additionally, calprotectin inhibited LPS-induced recruitment of PMNs in the rat air-pouch model of inflammation *in vivo* (211, 212). S100A9 differentially modified the responsiveness of neutrophils and dendritic cells to LPS, suggesting that the effects of calprotectin may be cell specific. While S100A9-deficient neutrophils exhibited a reduced secretion of cytokines (e.g., TNF-α and MCP-1) in response to LPS stimulation, inflammatory cytokine production in dendritic cells was exacerbated by S100A9 deficiency (213). Circulating concentrations of calprotectin increase with acute inflammation and during sepsis (214, 215), which has led some authors to suggest a proinflammatory role for this protein (216). Supporting this notion, Pepper and coworkers (217) showed that calprotectin plays a critical role during glomerulonephritis, amplifying autocrine and paracrine proinflammatory effects on BMDMs, renal endothelial cells, and mesangial cells. Indeed, calprotectin have an established clinical role as a biomarker in IBD (218).

In contradiction to these findings, anti-inflammatory, antinociceptive, and protective properties of calprotectin have also been described. In addition, regulation of S100A8 by GCs reinforces the idea of an anti-inflammatory role for this protein (219). For instance, calprotectin was suggested to be involved in the regulation of inflammatory processes in joints, since it produced marked anti-inflammatory and protective effects in models of adjuvant-induced arthritis in rats (220). Indeed, calprotectin deficiency was found in wound fluid from patients with non-healing venous leg ulcers, when compared with that from patients with healing open-granulating acute wounds (221). Sun and colleagues proposed protective and anti-inflammatory functions for calprotectin in sepsis. The authors showed that mice treated with S100A8 increased their survival rates and reduced tissue damage, inflammation, and oxidative injuries to major organ systems in a model of LPS-induced endotoxemia (222). Calprotectin was also shown to inhibit the oxidative metabolism of LPS-activated PMNs *in vitro*, which could contribute to reduce the oxidative organ injury seen in sepsis (223–225). Calprotectin suppressed NF-κB expression, proinflammatory cytokines, and inflammation in experimental autoimmune myocarditis (226), while the loss of calprotectin exacerbated T-cell activation and cardiac allograft rejection (227). In opposition, calprotectin aggravated post-ischemic heart failure through activation of RAGE-dependent NF-κB signaling (228). The diverging biological functions reported for calprotectin and its subunits suggest that their effects might be concentration dependent and influenced by the cellular and biochemical composition of the local milieu (229).

A novel and intriguing pro-resolving mechanism centered on neutrophils involves the generation of membrane borne microvesicles, also called microparticles or ectosomes (21). In 2004, Gasser and Schifferli (230) found that these microvesicles blocked the inflammatory response of macrophages exposed to zymosan and LPS. Further studies on neutrophil microparticles revealed that these microstructures could carry a variety of anti-inflammatory and pro-revolving mediators, enabling important modulatory functions in inflammation. Dalli and colleagues (231) defined the proteomic content of neutrophil microparticles. These authors observed that neutrophils have the ability to respond to a specific stimulus by producing microparticles loaded with a distinct proteomic profile, supporting the notion that microparticles production is a regulated process and might be endowed with very discrete functions (231). Some proteins, such as alpha-2-microglobulins, were identified to be selectively confined in vesicles generated from neutrophils adhered to an endothelial monolayer, whereas AnxA1 was more enriched in vesicles from exudate neutrophils. AnxA1 +ve vesicles possess anti-inflammatory properties (232) and allow the proper externalization of this pro-resolving mediator to gain access to extracellular surface receptors (i.e., FPR2) and to exert anti-inflammatory effects (39). AnxA1 acts as an exquisite brake for neutrophil adhesion to the microvascular wall, preventing over-exuberant cell transmigration to the inflammatory site (21, 233–235). We recently identified new properties for AnxA1 +ve vesicles, specifically those abundant in human synovial fluids collected from patients suffering from rheumatoid arthritis: these vesicles ensure the delivery of AnxA1 (and presumably other factors) to the chondrocyte in deep cartilage, enabling the activation of reparative circuits (236). In a recent review, we discussed the newly discovered modulatory roles of AnxA1 on neutrophil recruitment and other features of the resolution of inflammation (237).

### Distinct Macrophage Populations Mediate Acute Inflammation and Resolution Phases of Inflammation

Macrophages are one of the first cells to sense injury, infection, and other types of noxious conditions, triggering the immune response through the production of proinflammatory mediators (1). During resolution, macrophages play an anti-inflammatory role and are required for the clearance of apoptotic cells. Following efferocytosis, macrophages undergo a functional repolarization, switching from a pro- to an anti-inflammatory phenotype (238). Accordingly, efferocytosis is coupled with increased release of TGF-β and IL-10 and lower levels of proinflammatory cytokines, such as IL-6 (238–240). This change in the phenotype of macrophages also activates pro-resolving mechanisms, because they generate LXA_4_, which stimulates phagocytic activities without releasing proinflammatory mediators. This is an important non-phlogistic process typical of resolution, and shared, for instance, by GCs (241).

In addition to participating in the lipid mediator class switching discussed above, PGE_2_ is also important in macrophage reprogramming, mediating the transition from the acute to the resolution phase of inflammation. Early data from Kunkel’s group showed a suppressive effect of PGE_2_ on macrophage TNF-α and IL-1β production (242, 243), and this has been confirmed by other investigators (238). This inhibitory feature allows proinflammatory cytokines to regulate their own production using PGE_2_ as a self-induced modulator (242). Recently, MacKenzie and colleagues (244) reported that the addition of PGE_2_ to LPS-stimulated macrophages represses proinflammatory cytokine production but induces IL-10. In particular, PGE_2_ displayed a biphasic effect on IL-6 transcription: at early time points, this eicosanoid promoted IL-6 transcription but at later time points, it repressed the induction of IL-6 mRNA (244). Another study showed that PGE_2_ from activated bone marrow stromal cells promotes IL-10 in LPS-stimulated macrophages, an effect mediated by prostaglandin EP2 and EP4 receptors (245). PGE_2_ in combination with LPS was able to induce the mRNA for Arginase 1, LIGHT (TNFSF14), and SPHK1, potential markers of alternatively activated and regulatory macrophages (245), again suggesting long-lasting roles for this prostaglandin in macrophage reprogramming.

PGE_2_ has also been implicated in tissue maintenance and regeneration. This is supported by reports that indicate that increased levels of PGE_2_ were associated with increased regenerative capacity. In this regard, Zhang and colleagues showed that the inhibition of 15-hydroxyprostaglandin dehydrogenase (15-PGDH), a prostaglandin-degrading enzyme, potentiates tissue regeneration in multiple organs in mice. In a model of DSS-induced colitis, PGE_2_ elevation diminished colon ulcers, suppressed mucosal inflammation, and reduced colitis symptoms, in conjunction with increased cell proliferation in the DSS-damaged mucosa. Interestingly, the pharmacological induction of higher levels of PGE_2_ was associated with markedly increased rate and extent of liver regeneration in mice after partial hepatectomy as compared to control groups (246). In the lung, PGE_2_ is the major eicosanoid produced by fibroblasts, alveolar macrophages, and other lung cells, playing important roles in tissue repair processes and in immune-inflammatory response limitation (247). PGE_2_ directly inhibits several major pathobiologic functions of lung fibroblasts and myofibroblasts, including proliferation, migration, collagen secretion, and myofibroblast differentiation [reviewed in Ref. (248)]. Of note, diminished PGE_2_ production and/or signaling can be observed in human and animal lung fibrosis, reinforcing its relevance for proper resolution (249, 250).

### What the Future Reserves for Resolution

Undoubtedly, the inflammatory system is greatly complex. The history of the discovery of proinflammatory mediators reminds us that several decades of research were required to define the biology and pharmacology of the currently known mediators of inflammation. Since Sir Henry Dale and Patrick Laidlaw described some physiological effects of histamine *in vivo*, in 1910, immunological research has tremendously advanced (251). Pharmacological research has accompanied this progress, as historically represented by the discovery of antihistamines by Daniel Bovet and the identification of anti-H2R antagonists by Sir James Black, both awarded with the Nobel Prize in Physiology and Medicine (251). Subsequently, we made progress in the immunological and pharmacological fields of research, appreciating and shaping the concept of resolution of inflammation, and the mechanisms underpinning it. Fundamental concept here is the acceptance that resolution of inflammation is an active process evoked by specific classes of pro-resolving mediators, which differ from classical “anti-inflammatories” due to their ability to stimulate selective molecular and cellular programs of resolution. In the last decade, it has become evident that the enormous complexity of the proinflammatory system is mirrored at the level of pro-resolution pathways. Despite these remarkable advancements in the field, it seems that we have just started to scratch the surface of resolution mediators and other new cellular players are likely to be identified and defined in the near future. Likewise, we need to identify the major triggering pathway of these pro-resolving events, a phenomenon likely to be tissue- and/or disease-specific, as well as appreciate the complex networks among pro-resolving mediators. Such knowledge would be instrumental in developing pro-resolution based strategies to treat complex chronic inflammatory diseases in man, thus establishing a new area of pharmacology to be referred to as “resolution pharmacology” (22).

## Author Contributions

MS, LS, VP, MP, and MT conceived and wrote the manuscript, and realized the figure.

## Conflict of Interest Statement

The authors declare that the research was conducted in the absence of any commercial or financial relationships that could be construed as a potential conflict of interest. The reviewer VP and handling editor declared their shared affiliation, and the handling editor states that the process nevertheless met the standards of a fair and objective review.
